# Small Anther, a C_2_H_2_-Type Zinc Finger Transcription Repressor, Controls Rice Anther Length Through Regulation of Epidermal Cell Division

**DOI:** 10.3390/plants15152246

**Published:** 2026-07-23

**Authors:** Jiazhuo Wu, Yantong Liu, Yukang Xia, Ye Tao, Ling Zhao, Kai Lu, Wenhua Liang, Tao Chen, Cailin Wang, Yadong Zhang, Changjiang Zhao, Hongqiang An

**Affiliations:** 1 College of Agriculture, Heilongjiang Bayi Agricultural University, Daqing 163319, China; wjiajia0000@163.com; 2 Institute of Food Crops, Jiangsu Academy of Agricultural Sciences, Key Laboratory of Jiangsu Province for Agrobiology, East China Branch of National Center of Technology Innovation for Saline-Alkali Tolerant Rice, Nanjing 210014, China; 20240004@jaas.ac.cn (Y.T.); zhaoling@jaas.ac.cn (L.Z.); lukai@jaas.ac.cn (K.L.); whliang@jaas.ac.cn (W.L.); chentao@jaas.ac.cn (T.C.); clwang@jaas.ac.cn (C.W.); zhangyd@jaas.ac.cn (Y.Z.); 3 State Key Laboratory of Hybrid Rice, College of Life Sciences, Wuhan University, Wuhan 430072, China; 15704605354@163.com (Y.L.); xiayukang@kingfa.com.cn (Y.X.)

**Keywords:** rice (*Oryza sativa* L.), anther development, C_2_H_2_ zinc finger protein, transcriptional repressor

## Abstract

Anther length is a crucial agronomic trait that directly correlates with pollen number and pollination efficiency, thereby affecting rice grain yield and hybrid seed production. However, the specific molecular mechanisms regulating rice anther length remain largely unclear. In this study, we identified a *short anther* (*san*) mutant generated by CRISPR/Cas9, which exhibited significantly shortened anthers and reduced pollen number, without affecting other agronomic traits or pollen viability. Cytological observations revealed that the shortened anther phenotype in *san* mutants was caused by reduced epidermal cell number, rather than altered cell size. *SAN* was highly expressed in spikelets and developing anthers and encodes a nucleus-localized C1-1iG subclass C_2_H_2_ zinc finger protein with a conserved QALGGH motif and a C-terminal EAR motif. Further assays demonstrated that *SAN* functions as a transcriptional repressor, and could interact with the corepressor TPR2 in the nucleus. RNA-seq analysis indicated that *SAN* influences expression of genes associated with cell cycle and cytokinin, which are critical for epidermal cell division during anther development. Phylogenetic analysis showed that SAN was evolutionarily conserved in plants and was closely related to Arabidopsis SUP/ZFP11 and rice SRO. Collectively, our findings revealed that *SAN* specifically modulated rice anther length by promoting epidermal cell division and providing new insights into the molecular mechanism of anther size regulation.

## 1. Introduction

Rice (*Oryza sativa* L.) is a paramount staple crop worldwide, sustaining more than half of the global population. Grain yield, the most critical agronomic trait determining rice production potential, is fundamentally governed by plant reproductive success, especially efficient male reproductive development and subsequent fertilization. Anther development, as the central process of male gametophyte formation, directly determines pollen fertility, pollen quantity and fertilization efficiency, thus exerting a decisive impact on rice grain yield and final production [1,2]. Therefore, dissecting the molecular regulatory mechanisms underlying anther development is of great theoretical significance and practical application value for targeted improvement of rice reproductive traits and breeding of high-yield hybrid rice varieties.

Anther morphogenesis and maturation are highly sophisticated and orderly biological processes, involved meristematic cell differentiation, directional cell division, tissue patterning, tapetum development and pollen grain maturation [1,3]. A typical rice anther forms four distinct concentric somatic cell layers from outer to inner, namely the epidermis, endothecium, middle layer and tapetum, which encase and support the development of pollen mother cells and microspores, these somatic layers and reproductive cells coordinate with each other to ensure complete anther development and normal pollen formation [3,4,5,6]. Previous studies have confirmed that the proliferation and expansion of anther somatic cells are key determinants of final anther size, and anther length, as a core indicator of anther size, is closely positively correlated with pollen grain number and pollen release efficiency, further affecting pollination success and seed setting rate [7,8,9]. In general, plant organ size is controlled by the precise balance between cell proliferation and cell expansion and this regulatory mechanism is also conserved in anther development [10]. This process is orchestrated by a suite of transcriptional regulators, enzymes, and transporters that modulate fundamental pathways like sugar and lipid metabolism [3,4,5]. However, compared with the well-studied regulatory pathways of tapetum PCD, pollen wall formation and meiosis, the specific molecular mechanisms regulating anther size, especially anther length, in rice remain largely unclear, and the key functional genes involved in this process are rarely reported [11].

In hybrid rice breeding, both the three-line and two-line systems highly depend on fertile restorer lines to provide sufficient and viable pollen for male sterile lines to ensure high hybrid seed production yield and seed purity. Anther length is positively correlated with pollen amount—long anthers can accommodate more pollen grains—which effectively improves the pollination efficiency of restorer lines and increases the yield of hybrid seed production. For example, introducing rye chromosome 4R into common wheat can significantly increase anther length and pollen grain number, significantly improving the outcrossing potential of wheat, which fully proves that manipulating anther size is a feasible strategy for crop reproductive trait improvement [8]. Therefore, identifying and utilizing key genes that regulate rice anther length is of great practical significance for optimizing rice restorer lines and enhancing the efficiency of hybrid rice seed production.

C_2_H_2_-type zinc finger proteins (ZFPs) represent the largest family of transcriptional regulators in plants, involved in diverse processes from development to stress responses [12]. According to the number and arrangement of cysteine and histidine residues involved in zinc ion coordination, plant C_2_H_2_ ZFPs can be divided into multiple subfamilies including A1, A2, A3, A4, B1, C1, C2 and C3 [13,14]. Among them, the C1 subgroup of C_2_H_2_ ZFPs is characterized by the highly conserved QALGGH motif in the zinc finger domain, and this subgroup plays an irreplaceable role in plant floral organ development [14]. In the model eudicot *Arabidopsis thaliana*, multiple C1 subgroup C_2_H_2_ ZFPs have been proven to be key regulators of flower development, such as SUPERMAN (SUP) and RABBIT EARS (RBE). These two proteins act as transcriptional repressors, relying on the C-terminal ethylene-responsive element-binding factor-associated amphiphilic repression (EAR) motif to inhibit the expression of floral homeotic genes, maintaining the boundary between floral organs and determine floral meristem fate [15,16]. In rice, the C_2_H_2_ ZF transcription factor SMALL REPRODUCTIVE ORGANS (SROs), an ortholog of Arabidopsis SUP, is involved in the regulation of stamen filament development and floral meristem determinacy and functions downstream of B- and C-class floral organ identity genes, indicating the functional conservation of C1 subgroup C_2_H_2_ ZFPs in plant reproductive development.

Nevertheless, critical research gaps remain in the current understanding of rice anther developmental regulation: few currently identified anther regulatory genes specifically modulate anther length via modulating epidermal cell proliferation, whereas most characterized anther regulators trigger pleiotropic developmental phenotypes or complete male sterility in rice. Furthermore, the transcriptional link between EAR motif repressors, cytokinin signaling and early anther cell cycle remains poorly defined. Although a small number of rice C_2_H_2_ ZFPs have been reported to regulate stress tolerance, plant height and tiller development [17,18], the functions of most C_2_H_2_ ZFPs in anther development, especially in the control of anther size and length, remain largely unknown [19].

In this study, we identified a *short anther* (*san*) mutant in rice, which specially modulated the length of anther without affecting other agronomic traits and pollen viability. Further investigations showed that the reduced epidermal cell division was the primary reason for the shortened anther in *san* and the cytokinin and cell cycle pathway associated with cell proliferation may have contributed to the insufficient cell division. This study provides new insights into the molecular mechanism of anther size regulation in rice and offers a potential genetic resource for breeding of high-pollen-yield restorer lines in hybrid rice.

## 2. Results

### 2.1. Generation and Identification of the SAN Mutant

**Figure 1:**
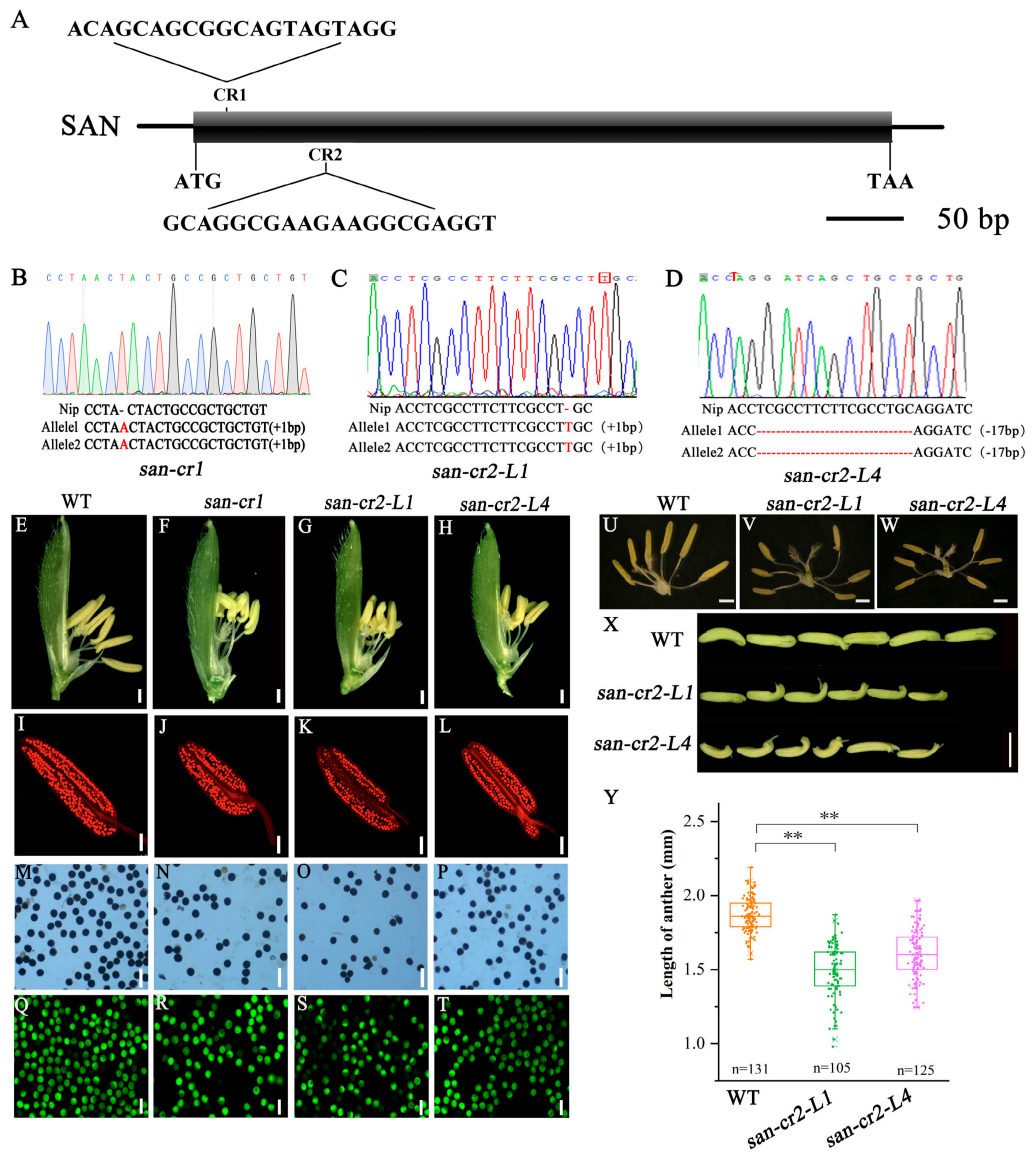
Identification and phenotype analysis of rice *small anther* (*san*) mutants. (**A**) Schematic diagram of the *SAN*-*CR* target sites in rice. Both target sites are 20 bp in length, and the dark-colored regions represent the coding region sequences of *SAN*. (**B**–**D**) Sequencing peak profiles of rice *san*-*cr1* and *san*-*cr2* mutant. (**E**–**H**) Spikelet and anther morphology of the WT and *san*-*cr1*, *san*-*cr2*-*L1*, *san*-*cr2*-*L4* mutants. Scale bar = 1 mm (**I**–**L**) Observation of pollen development in WT and *san* mutants through eosin B whole-mount clearing staining. Scale bar = 500 μm. (**M**–**P**) Observation of pollen grains from WT and *san* by I_2_-KI staining. Scale bar = 100 μm. (**Q**–**T**) Observation of pollen viability in WT and *san* through FDA staining. Scale bar = 100 μm. (**U**–**W**) Isolated mature pistils and stamens from florets of WT, *san*-*cr2*-*L1* and *san*-*cr2*-*L4*. Scale bar = 1 mm. (**X**,**Y**) Observation and statistical analysis of anther lengths in WT and *san* mutant. Scale bar = 2 mm. *n* indicates the number of anthers examined. ** represents *p* < 0.01 with highly significant difference.

To investigate the function of rice *SAN* (*LOC_Os02g01090*), we constructed two CRISPR/Cas9 knockout vectors (*SAN*-*CR1* and *SAN*-*CR2*), targeting its exon region, and generated T_0_ transgenic plants via two independent *Agrobacterium*-mediated transformation events. Sequencing of the target sites revealed multiple editing events in the T_0_ generation. Through multi-generational genetic segregation and molecular screening, we obtained transgene-free homozygous mutants with three stable inheritance patterns: *san*-*cr1, san*-*cr2*-*L1* and *san*-*cr2*-*L4* (Figure 1A). Sequence analysis confirmed that *san*-*cr1* carried a single adenine (A) insertion (Figure 1B), *san*-*cr2*-*L1* harbored a single thymine (T) insertion (Figure 1C), and *san*-*cr2*-*L4* contained a 17 bp deletion (TCGCCTTCTTCGCCTGC) (Figure 1D). SnapGene-based protein sequence prediction demonstrated that these insertions (*san*-*cr1*, *san*-*cr2*-*L1*) and deletion (*san*-*cr2*-*L4*) induced frameshift mutations, leading to premature termination codons at amino acid position 39.

### 2.2. SAN Mutants Exhibit Reduced Anther Length Without Affecting Other Agronomic Traits

Phenotypic characterization revealed no significant differences in plant height between *san* mutants and wild-type (WT) plants at 7, 14, or 90 days after germination (Figure S2A–I) and the tiller number was comparable at 90 DAP (Figure S2J). These results indicated that loss of *SAN* function does not impact vegetative development. During reproductive development, the panicle length, primary branch number, secondary branch number, and seed setting rate were also unchanged in *san* mutants relative to WT (Figure S3A–G). Microscopic dissection of floral organs revealed that the pistils of the *san* mutants developed normally with paired feathery stigmas, and stamens formed six anthers, which were consistent with WT, but the anther length of *san* was significantly shorter than that of WT (Figure 1E–L,U–Y).

To further explore the phenotypic characteristics of the shortened anther in *san* mutants, two individual lines *san*-*cr2*-L1 and *san*-*cr2*-L4 were used for observation and statistical analysis. (Figure 1U–X). The statistical results showed there were extremely significant differences in anther length between *san* mutants and WT, where the mature anther length of the *san*-*cr2*-*L1* and *san*-*cr2*-*L4* line was 25.5% and 13.3% shorter than that of the WT, respectively (Figure 1Y). To explore whether the shortened anther phenotype affected pollen maturity and viability, I_2_-KI staining (Figure 1M–P) and FDA staining (Figure 1Q–T) were performed. The results showed the pollen maturity and viability of *san* mutants were similar to WT, but the number of pollen in *san* mutants were fewer than WT. These findings indicated that *SAN* specifically regulated anther development, reducing anther length and pollen number without compromising pollen fertility or other agronomic traits.

### 2.3. SAN Modulates Anther Growth by Regulating Epidermal Cell Division

**Figure 2:**
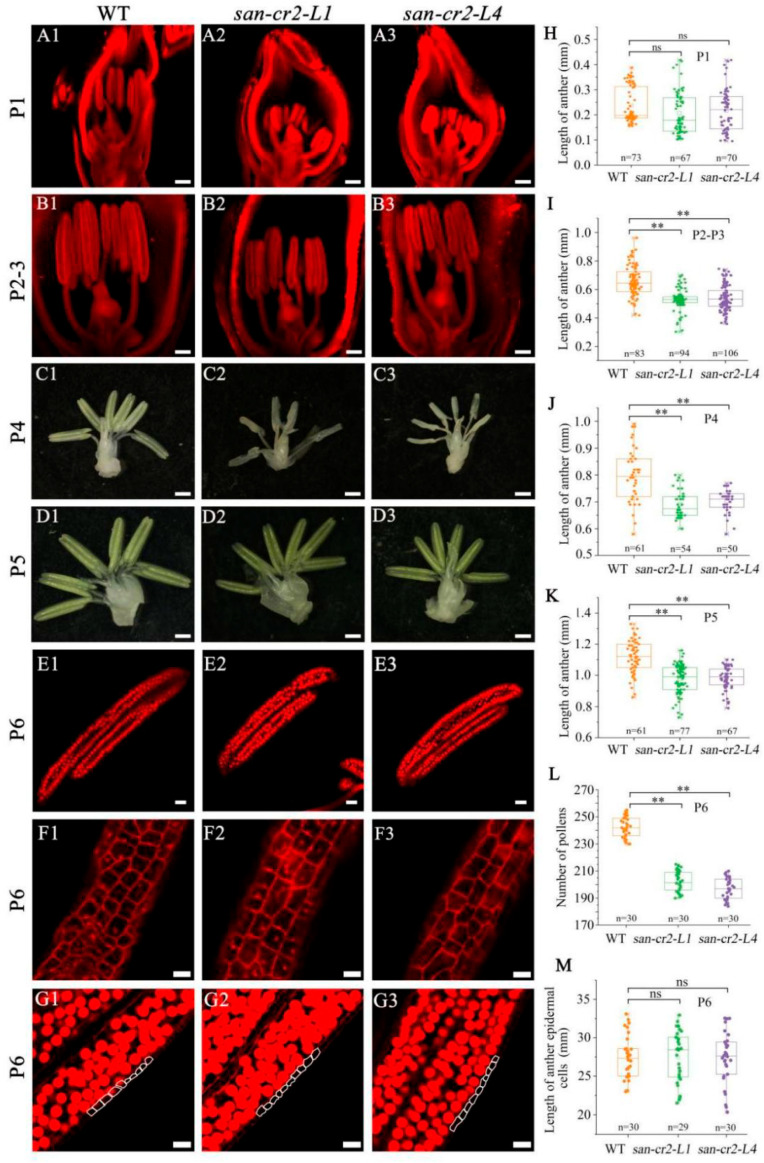
Phenotypic characteristics and length statistics of the anther in rice *san* mutants. (**A1**–**D3**) Observation of anther length during P1–P5 stages of panicle development in WT and *san* mutants. Scale bars = 500 μm; (**E1**–**E3**) number of pollens in P6 stage. Scale bars = 200 μm. (**F1**–**G3**) Observation of anther epidermal cell from WT and *san* mutants. Scale bar = 25 μm (**F1**–**F3**), scale bar = 75 μm (**G1**–**G3**). (**H**–**M**) Statistical analysis of anther length, pollen number, epidermal cell length of anthers in WT and *san* mutants. *n* indicates the number of anthers observed (**H**–**L**) and the number of anther epidermal cells (**M**). ns represents no significant difference; ** represents *p* < 0.01 with highly significant difference.

To determine the developmental stage at which anther shortening initiated in *san* mutants, panicle tissues from WT and *san* mutants at stages P1, P2–P3, P4, P5, and P6 were collected (Figure 2(A1–E3)). Due to the small size and difficulty in isolating anthers at stages P1 and P2–P3, entire spikelets from these stages were cleared and stained for structural observation (Figure 2(A1–B3)), while glumes were removed for analysis of more developed anthers at P4 and P5 stages (Figure 2(C1–D3)). Anther length at different developmental stages was measured and analyzed using Digimizer 6.2.0 software. At stage P1, no significant difference in anther length was observed between the mutants and the WT (Figure 2(A1–A3),H). At stage P2–P3, anther length decreased by 26.1% in *san*-*cr2*-*L1* and 21.3% in *san*-*cr2*-*L4* (Figure 2(B1–B3),I). At stage P4, the reduction was 20.3% in *san*-*cr2*-*L1* and 16.5% in *san*-*cr2*-*L4* (Figure 2(C1–C3),J). At stage P5, anther length in *san*-*cr2*-*L1* and *san*-*cr2*-*L4* mutants was reduced by 14.7% and 14.0%, respectively, compared to the WT (Figure 2(D1–D3),K). The pollen quantity at the mature stage of P6 was examined and the result revealed a significant reduction of 19.7% in *san*-*cr2*-*L1* and 22.9% in *san*-*cr2*-*L4* (Figure 2(E1–E3),L), which corresponds closely to the extent of anther length reduction. These results suggested that *SAN* was required for normal anther morphological development starting at the P2–P3 developmental stage and loss of AES led to the reduced pollen number in mature anther of *san*.

To investigate whether the shortened anther phenotype in the mutant resulted from reduced cell size or decreased cell number during anther development, mature anthers from both WT and *san* mutant were cleared and stained with eosin B. The stained anthers were observed using confocal microscopy to measure the length and count the number of epidermal cells in WT and *san* mutant anthers (Figure 2(F1–G3),M). The results indicated no significant difference in the average length of anther epidermal cells between the *san* mutant and WT (Figure 2M). In summary, the *SAN* likely affected epidermal cell division during rice anther development, leading to a reduced number of epidermal cells and consequently shorter anthers in the *san* mutant.

### 2.4. SAN Is Preferentially Expressed in Developing Anthers

To investigate the spatiotemporal expression pattern of the *SAN* across various organs and developmental stages of rice, we first analyzed microarray data from the SIGNAL database (http://signal.salk.edu/ (accessed on 20 March 2021)) and the BAR database (http://bar.utoronto.ca/ (accessed on 20 March 2021)), followed by experimental validation via quantitative real-time PCR (qRT-PCR). Microarray database analysis showed that the *SAN* was expressed in all examined tissues, with higher expression levels in stages P1, P4, P5, and mature leaves (Figure S4A). Similarly, the BAR database indicated ubiquitous expression of *SAN* across multiple tissues, with notably high expression in stages P1, P4, P5, S4, and S5 (Figure S4B).

**Figure 3:**
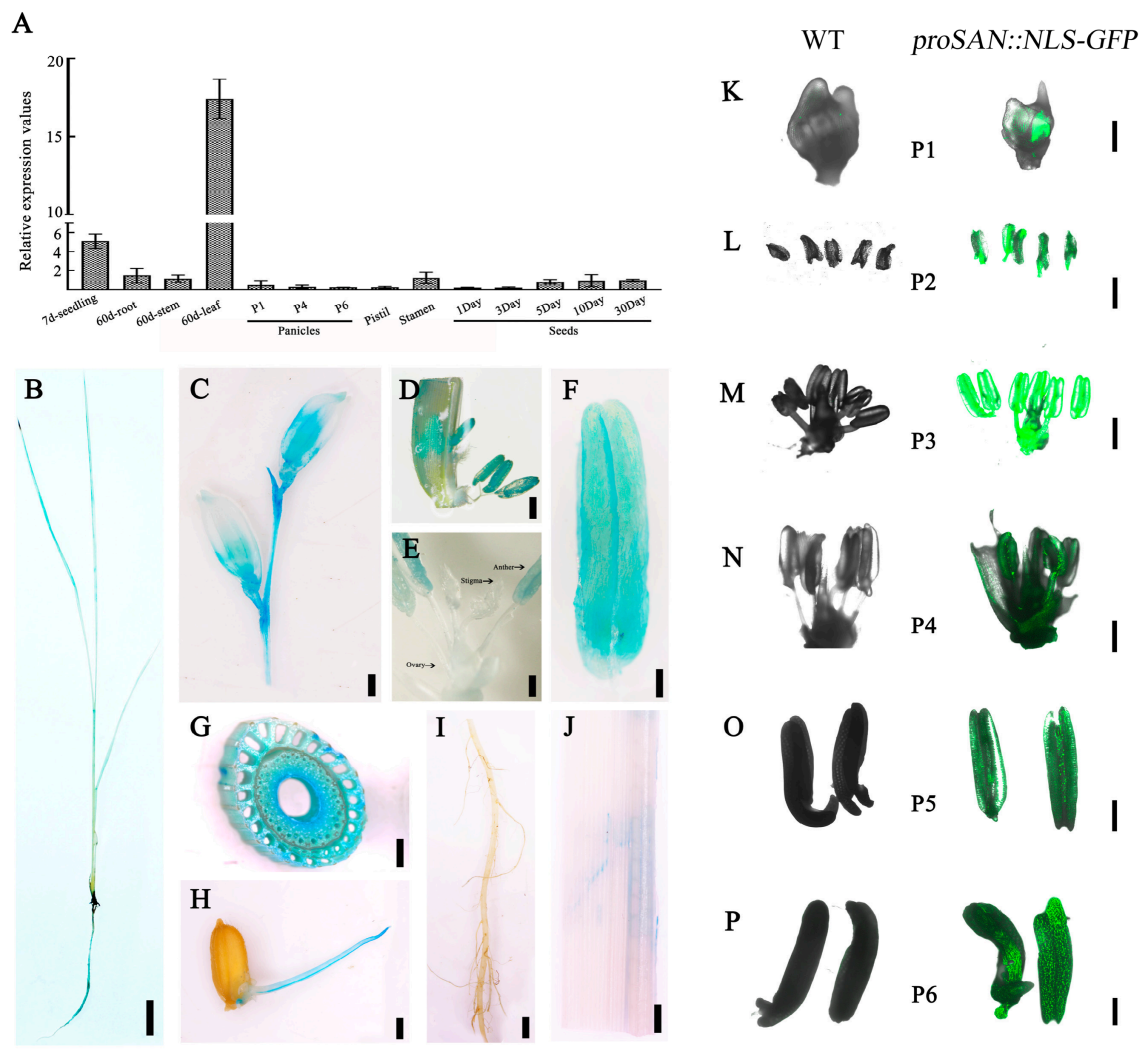
Expression profile of the *SAN* in various rice tissues. (**A**) Expression level of the *SAN* in different tissues detected through qRT-PCR. (**B**–**J**) *SAN* expression analysis through GUS staining in different tissues. (**B**) The 14-day-old seedling. Scale bar = 2 cm (**C**) Panicle at the booting stage. Scale bar = 1 mm (**D**) Mature floret. Scale bar = 1 mm (**E**) Mature pistil. Scale bar = 1 mm (**F**) Mature anther. Scale bar = 0.2 mm (**G**) Stem of a 60-day-old plant. Scale bar = 1 mm (**H**) Seed 2 days after germination. Scale bar = 1 mm (**I**) Root of a 60-day-old plant. Scale bar = 3 mm (**J**) Leaf of a 60-day-old plant. Scale bar = 3 mm (**K**–**P**) Observation of *SAN* expression in anthers during stages P1–P6 via GFP fluorescence. Scale bars = 500 μm.

The expression pattern of *SAN* in different tissues analyzed through qRT-PCR showed that *SAN* was expressed in all examined tissues, with the highest expression levels in 60-day-old leaves, followed by 7-day-old seedlings and stamens (Figure 3A). Transgenic rice plants carrying the *proSAN::GUS* reporter were analyzed by histochemical staining. GUS activity was visible in leaves and roots of 14-day-old seedlings, emerging shoots of 2-day-old germinating seeds, the vascular tissues of stems of 60-day-old plant and strongly detected in young spikelets, especially in developing anthers but weakly detected in mature pistils, leaves and roots in 60-day-old plants (Figure 3B–J). To further examine the expression pattern of *SAN* in anther, GFP signals were detected in the anthers of *proSAN::NLS*-*GFP* transgenic plant from P1–P6 development stages. *SAN* was ubiquitously expressed across all the developmental stages of anther from P1–P6, and with no signals in WT controls (Figure 3K–P). These results confirmed that *SAN* was widely expressed in rice, with preferential expression in spikelets and developing anthers, consistent with its role in anther development. The highest expression of *SAN* in leaf indicated it may play important role in leaf, but could be functional redundancy with other genes as the *san* mutant showed no defects in vegetative stage. In summary, the anther enriched expression feature of *SAN* was consistent with its core biological role in regulating rice anther elongation.

### 2.5. SAN Is a Nuclear-Localized Protein

**Figure 4:**
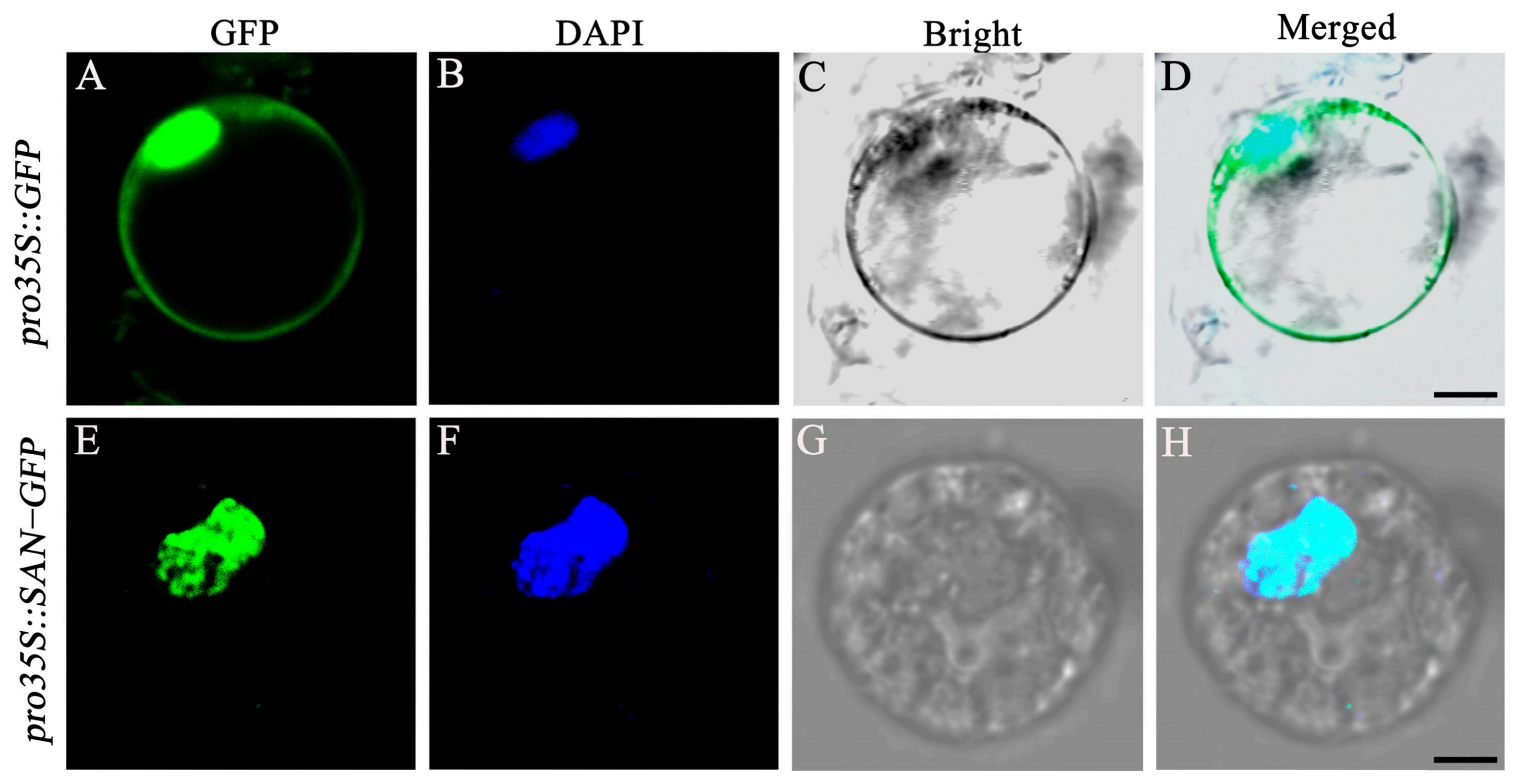
Subcellular localization of SAN protein in rice. (**A**–**H**) Subcellular localization of SAN in rice protoplasts by *pro35S::GFP* and *pro35S::SAN*-*GFP* transient transformation. *pro35S::GFP* served as the negative control (**A**–**D**). Colocalization of *pro35S::SAN*-*GFP* with the DAPI signals (blue) in the nucleus (**E**–**H**). GFP, green fluorescence protein. Bars = 15 μm.

To investigate the subcellular localization of SAN, we observed the rice protoplasts expressing *pro35S::GFP* and *pro35S::SAN*-*GFP* by transient transformation. When the control *pro35S::GFP* vector was expressed, the green fluorescent signal was diffusely distributed across the cytoplasm and nucleus (Figure 4A–D). In contrast, expression of the *pro35S::SAN*-*GFP* fusion protein resulted in a fluorescent signal that co-localized with DAPI-stained nuclei (Figure 4E–H), confirming that the SAN protein was localized to the nucleus.

### 2.6. SAN Regulates the Expression of Cell-Division-Related Genes

**Figure 5:**
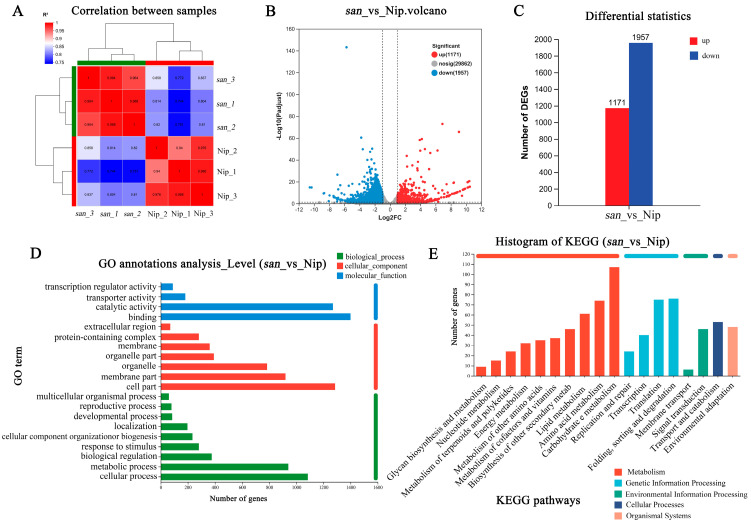
Gene Ontology (GO) and Kyoto Encyclopedia of Genes and Genomes (KEGG) analysis in the transcriptome of WT and *san*. (**A**) Correlation analysis of transcriptomic data between WT and *san*. (**B**) Volcano plot analysis of differentially expressed genes (DEGs) identified from WT versus *san*. Adjusted *p*-values < 0.00001 are displayed as 0.00001. Dashed lines represents the no-significant genes. (**C**) The number of DEGs between WT and *san*, filtered by a 2-fold change threshold and an adjusted *p*-value < 0.05. (**D**) GO categorization based on DEGs. (**E**) KEGG pathway enrichment analysis based on DEGs between WT and *san*.

To elucidate the molecular mechanisms by which *SAN* regulated anther development, we performed RNA-seq analysis of mature anthers from WT and *san*-*cr2*-*L1* mutants. Correlation and volcano plot analyses confirmed significant divergence in gene expression profiles, with 1171 upregulated and 1957 downregulated genes in *san* mutants (Figure 5A–C). To explore which biological processes were affected in *san*, Gene Ontology (GO) enrichment analysis and Kyoto Encyclopedia of Genes and Genomes (KEGG) pathway analysis of the differentially expressed genes (DEGs) were conducted. GO enrichment analysis of all DEGs revealed significant enrichment in binding, catalytic activity, cell/membrane part, and cellular/metabolic processes (Figure 5D). KEGG pathway enrichment analysis showed that carbohydrate metabolism, amino acid metabolism, lipid metabolism, folding, sorting and degradation, translation, signal transduction, transport and catabolism and environmental adaptation were affected in *san* mutant (Figure 5E).

**Figure 6:**
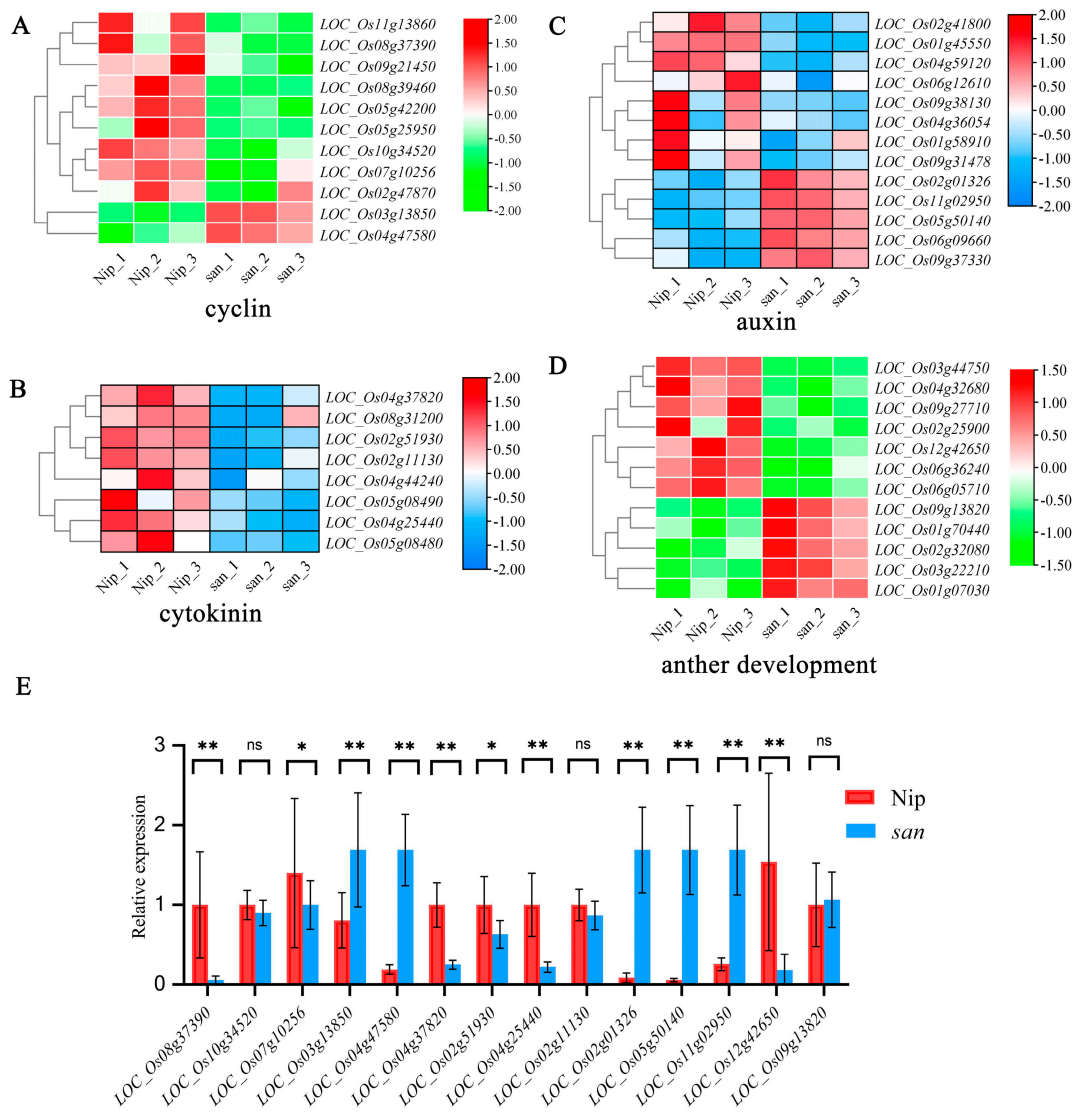
Expression analysis of anther development and hormone-associated genes by heatmap. (**A**–**D**) Expression analysis of gene related to cyclin (**A**), cytokinin (**B**), auxin (**C**) and anther development (**D**). Color scale represents the expression levels, which were obtained log_2_ value of TPM of transcriptome analysis. (**E**) Relative expression of gene related to cyclin, cytokinin, auxin and anther development detected through qRT-PCR. Values are presented as mean ± SE. Three biological replicates and three technical replicates were used for each gene expression analysis. Significant differences were identified using Student’s *t*-test at *p* < 0.01. ns represents no significant difference; * represents *p* < 0.05 with significant difference. ** represents *p* < 0.01 with highly significant difference.

Given the reduced epidermal cell number in *san* mutant anthers, we analyzed the expression of cell division and growth related genes via heatmap using transcriptome data from WT and *san* mutants (Figure 6A–D). We focused on the key biological pathways, such as cyclin, auxin and cytokinin and anther development, and the heatmaps of normalized expression levels revealed that the cyclin and cytokinin was most severely perturbed. The core cell cycle regulators, including cyclin-dependent kinases and cyclin genes, displayed severe expression suppression, which directly correlated with the reduced cell division number observed in *san* anthers (Figure 6A). The down-regulation of the cytokinin pathway further weakened the upstream signal promoting cell proliferation, aggravating insufficient cell division together with the cell cycle pathway (Figure 6B). The auxin pathway was partial up-regulated and down-regulated, which ensure the balance of auxin in the development of anther and the cell size was unchanged in *san* mutant (Figure 6C). In contrast, the anther-specific developmental pathway also maintained a balanced expression pattern with partial up-regulation and partial down-regulation (Figure 6D). The expression of the genes analyzed in the transcriptome were also confirmed through the qRT-PCR (Figure 6E). This transcriptional stability ensured that all processes required for pollen development and fertility remain unimpaired, explaining why the *san* mutant produced fully viable pollen despite its short anther phenotype. In summary, the reduced anther in *san* mutant influenced the expression of genes associated with cell proliferation pathways, while stable expression of pollen development genes preserves fertility.

### 2.7. SAN Encodes a C1-liG Subclass C_2_H_2_ Zinc Finger Transcription Factor

**Figure 7:**
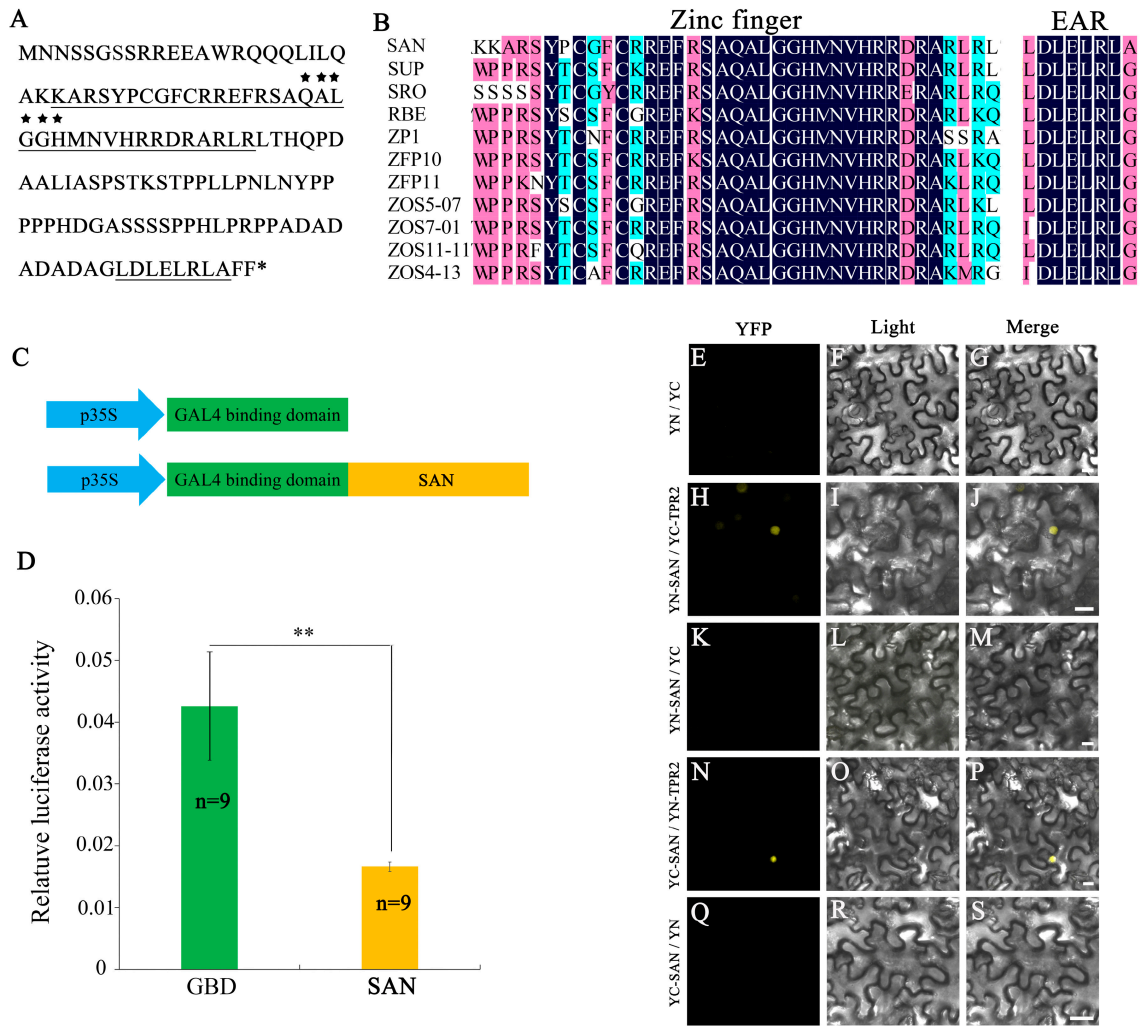
SAN functioned as transcriptional repressor and interacted with TPR2. (**A**) The amino acid sequence of SAN protein. * represents the QALGGH motif. The underlines represent Zinc finger and EAR domain respectively. (**B**) The alignment and domains analysis of SAN and its homologous proteins. (**C**) The schematic diagram of effector constructs for dual-luciferase reporter assay. (**D**) Measurement of relative luciferase activity of *GBD* and *GBD*-*SAN*. ** represents highly significant difference, *p* ≤ 0.01. (**E**–**S**) SAN interacted with TPR2 examined by BiFC assays in the epidermis cells of tobacco. YFP^N^, the N-terminal region of yellow fluorescent protein. YFP^C^, the C-terminal region of yellow fluorescent protein. YFP, yellow fluorescent protein indicates that the two proteins can interact with each other. Bars = 25 μm.

The C1 subgroup defined by the conserved QALGGH motif at positions 2–7 of the α-helix was one of the largest family in C_2_H_2_ zinc finger proteins and contain one to five zinc finger domains. Based on the number of QALGGH motif, the C1 family is subdivided into five subfamilies: C1-1i (33 members), C1-2i (20 members), C1-3i (8 members), C1-4i (2 members), and C1-5i (1 member) [19]. The sequence analysis of SAN protein showed it had a QALGGH motif in N-terminal region and an EAR motif in C-terminal region (Figure 7A), confirming its classification as a C1-liG subclass C_2_H_2_ ZFP.

### 2.8. Phylogenetic Analysis of SAN and Its Homologs

The C1-liG subgroup includes six members in rice (SAN, SRO, ZOS7-01, ZOS5-07, ZOS11-11, ZOS4-13) and five members in *Arabidopsis* (SUP, RBE, ZP1, ZFP10, ZFP11). Amino acid sequence alignment of C1-liG subgroup proteins by DNAMAN (Figure 7B) revealed three conserved motifs, indicating functional conservation across orthologs. Phylogenetic analysis showed that *Arabidopsis* RBE and ZFP10 were closely related to rice ZOS5-07, ZOS11-11, and ZOS7-01 (Figure S5A). The *Arabidopsis* ZP1 clustered with rice ZOS4-13 and *Arabidopsis* SUP/ZFP11 shared the closest evolutionary relationship with rice SRO/SAN (Figure S5A). The results showed that SAN shared the more closer relationship with SUP and SRO, which was consistent with their function in the development of anther as the previous study [20].

Phylogenetic analysis based on SAN and its homologous proteins from various species showed that SAN was widely distributed in monocotyledon, dicotyledon, moss and animals. SAN exhibited strong evolutionary conservation in monocotyledon and dicotyledon, which clustered with *Marchantia polymorpha*, *Sorghum bicolor*, *Oryza brachyantha*, *Aegilops tauschii* subsp, *Strangulata* and showed closest phylogenetic relationship with *Oryza brachyantha* and *Aegilops tauschii* subsp. In contrast, the SAN was phylogenetically distant from its homologs in *Homo sapiens*, *Mus musculus* and *Drosophila sechellia*, indicating evolutionary divergence between plant and animal ZFPs (Figure S5B).

### 2.9. SAN Acts as a Transcriptional Repressor and Interacts with TPR2

*SAN* encoded a transcription factor of C_2_H_2_ type and, to further explore the function of *SAN*, the transcriptional activity of SAN was conducted via dual-luciferase reporter assay in tobacco leaves. Compared to the control, co-expression of 35S::GAL4BD-SAN significantly repressed reporter gene activity (Figure 7C,D), indicating that SAN functioned as a transcriptional repressor, likely mediated by its C-terminal EAR motif.

Since EAR motifs typically recruit transcriptional corepressors such as TPL/TPR proteins, we used the STRING database to predict potential proteins that could interact with SAN and identified TPR2 as the candidate protein. Bimolecular fluorescence complementation (BiFC) assays in *Nicotiana benthamiana* leaves were performed to detect the interaction between SAN and TPR2. Specific yellow fluorescent signals were observed in the nucleus of epidermal cells when co-expressing SAN-YFP^N^/TPR2-YFP^C^ or SAN-YFP^C^/TPR2-YFP^N^ constructs (Figure 7E–S), but not in the corresponding negative controls (Figure 7E–G,K–M,Q–S). These results confirmed that SAN exhibits transcriptional repression activity and physically interacted with the TPR2 corepressor in the nucleus.

## 3. Discussion

### 3.1. SAN Specifically Regulates Anther Length by Modulating Epidermal Cell Division

Anther development is a complex process regulated by multiple pathways, including hormonal signaling, phosphorylation cascades, and ubiquitination-mediated protein degradation [21,22,23]. Previous studies in rice and *Arabidopsis* have shown that mutations affecting anther development often trigger pleiotropic defects in vegetative growth, floral organ identity, or pollen fertility, complicating the functional characterization of genes specifically dedicated to anther size regulation [20,24]. In contrast, the *san* mutant generated in this study exhibited a highly specific phenotype, only anther length was drastically reduced, with no pleiotropic effects on agronomic traits. Notably, pollen viability and maturity were unaffected in *san* mutants, with only pollen grain number decreased proportionally to anther length reduction. Spatiotemporal analysis of anther developmental stages revealed that anther shortening in *san* mutants initiates at the P2–P3 stage, with significant length differences detectable from this stage to mature anthers. Cytological observations further confirmed that SAN regulates anther growth exclusively by promoting epidermal cell division during early anther development, rather than influencing cell expansion.

This mode of action distinguishes *SAN* from previously reported anther regulators in rice, many of which target tapetum programmed cell death, pollen wall formation, or meiosis, and highlights its unique role in cell proliferation control during early anther morphogenesis. Research has shown that C_2_H_2_ zinc finger protein SRO influenced the development of pistil and stamen. The loss of SRO led to the shortened anther length, accompanied by reductions in both cell size and number [20]. The study suggested that the decrease in floral organ size was associated with plant hormones and demonstrated that SRO can interact with the auxin response factor IAA19 [20]. Plant organ size was primarily governed by coordinated cell proliferation and cellular expansion. The initial proliferative cascade facilitated substantial cellular multiplication, while the later expansive cascade mediated the volumetric growth of individual cells until these cells reached their functionally matched physiological dimensions [25,26]. Among the core regulatory factors, auxin, cytokinin, and cell cycle signaling pathways served as central modulators that fine-tune both cellular proliferative and expansive behaviors during organ size formation [26]. For example, the OsMAPK6 encoded mitogen-activated protein kinase, and *osmapk6* mutant exhibited small anthers and reduced fertility. Mutations in ARF6/8, which were involved in auxin signaling, caused a reduction in the size of flower organs [27]. The *AtERF19* gene regulated meristem activity and flower organ size through the regulation of genes involved in CLAVATA-WUSCHEL (CLV-WUS) and auxin signaling [28].

Floral organ morphogenesis and final organ size were predominantly governed by cell-cycle-mediated cell proliferation and post-mitotic cell expansion [29,30]. Numerous studies have confirmed that the duration of cell division phase and cell cycle progression rate in floral primordia were the primary determinants of anther, petal and pistil final morphological size [31]. In rice reproductive development, anther size was largely defined by the proliferation activity of mitotic precursor cells at early P1–P6 anther developmental stages, where G1/S and G2/M phase transition of the cell cycle dominated anther primordial cell number accumulation [32]. Transcription factors that modulated floral organ growth frequently regulated organ size by targeting cyclin family genes or cell cycle checkpoint factors to alter cell proliferation kinetics [33]. Combined with our experimental results, we speculated that the *SAN* may participate in modulating rice anther cell division via fine-tuning cell cycle progression in developing anther tissues. Consistent with the consensus regulatory model, the preferential expression of *SAN* in early-stage developing anthers may maintain normal cell division activity of anther wall cells, thereby determining the final anther length.

Here, we identified *SAN* as a key regulator of anther length in rice. Loss-of-function in *SAN* led to shorter anthers due to a reduction in the number of epidermal cells, without affecting cell size or pollen viability. Transcriptomic analysis of *san* mutant anthers revealed extensive transcriptional reprogramming, with over 3000 differentially expressed genes (DEGs) identified compared to WT. GO and KEGG enrichment analyses showed that these DEGs were significantly enriched in pathways related to cell division, cell wall biosynthesis, hormone signaling, and carbohydrate metabolism, consistent with the observed defects in cell proliferation and anther growth. Notably, cyclin- and cytokinin-related genes were the most severely down-regulated in *san* mutant, which further corroborated that *SAN* primarily controlled cell division rather than cell expansion. The genes associated with auxin and anther development showed partial up-regulated and down-regulated, implying a potential compensatory transcriptional program that maintained anther development, preserved normal anther cell size, and ensured unimpaired pollen fertility. Collectively, these transcriptomic changes indicated that loss of *SAN* influenced the expression of genes related to cyclin and cytokinin, indicating that *SAN* may function in anther length development through cell proliferation pathway.

### 3.2. SAN Encodes a Conserved C1-liG Subclass of C_2_H_2_ Zinc Finger Proteins

The *SAN* encoded a zinc finger protein containing only a single zinc finger domain and an EAR motif, thereby classifying SAN within the C1-1iG subgroup of C_2_H_2_ zinc finger proteins [34]. This subgroup comprised well-characterized transcriptional repressors. In rice, it consisted of six members: SAN, SRO, ZOS7-01, ZOS5-07, ZOS11-11, and ZOS4-13. In *Arabidopsis*, this protein group included five members: SUP, RBE, ZP1, ZFP10, and ZFP11 [35]. Sequence alignment and phylogenetic analysis of these 11 proteins revealed that SAN shared closer similarity with SUP and ZFP11 from *Arabidopsis* and SRO from rice. Since previous studies have indicated that SRO was evolutionarily homologous to SUP [35], it was inferred that SAN was more likely to be orthologous to ZFP11 in *Arabidopsis*. Notably, SAN, along with the previously characterized SUP, SRO and RBE, participated in rice anther developmental progression. However, obvious functional divergence exists among these regulatory genes. Distinct from SUP, SRO and RBE, which globally modulate whole floral organ morphogenesis and profoundly affect pollen fertility [20,35], SAN specifically controlled anther longitudinal elongation with no detectable alterations in other agronomic traits. Interestingly, although *SAN* was broadly expressed in multiple tissues, the mutant phenotype was primarily confined to anther length. Unlike previously reported regulators that simultaneously affect floral organ identity, tapetum development, pollen maturation or fertility, *SAN* appeared to specifically regulate anther elongation without compromising pollen viability, suggesting a distinct regulatory mechanism. This observation may be attributed to functional redundancy among other members of the C1-1iG subgroup in rice, which likely compensate for the loss of SAN function in other organs. These phenotypic and regulatory characteristics further demonstrated that SAN mediated anther development via a unique and unreported molecular regulatory cascade, which was fundamentally different from the regulatory modes of SUP, SRO and RBE.

### 3.3. SAN Functions as a Transcription Repressor

Subcellular localization assays confirmed that SAN was exclusively targeted to the nucleus, consistent with its role as a transcription factor. Dual-luciferase reporter assays in tobacco leaves demonstrated that SAN significantly repressed reporter gene expression, verifying its function as a transcriptional repressor. Transcription repression complexes, formed by a repressor and its corepressors, were essential for plant development. EAR motif-containing transcriptional repressors are widespread in plants and typically exert their regulatory effects by recruiting corepressor proteins, such as TOPLESS (TPL) and TOPLESS-RELATED (TPR) family members, to form repressor complexes that inhibit the transcription of downstream target genes [36,37]. TPL/TPR proteins functioned as conserved corepressors of the Groucho/Tup1 family, which do not bind DNA directly but are tethered to genomic loci via their interaction with the EAR motif of partner transcription factors [36]. The corepressors TPL cooperated with histone deacetylases (HDACs) to remodel chromatin, switching it from active into inactive state [38,39,40]. In the jasmonate signaling pathway, JAZ proteins inhibited the transcription factor MYC2. This repression relied on the adaptor protein NINJA, whose C-terminal EAR motif recruited TPR corepressors to suppress MYC2-mediated transcription. As a transcriptional repressor, NINJA worked together with TPR corepressors to negatively regulate jasmonate responses [41]. This indicated that the EAR motif was a key functional module for transcriptional repression in jasmonate signaling pathways. Its repressive function typically relied on recruiting TPR-class corepressors, which subsequently recruited histone-modifying enzymes such as deacetylases [42] and chromatin-remodeling associated histone methyltransferases [43], thereby executing transcriptional repression.

The SAN contained a conserved EAR motif and interacted with the rice corepressor TPR2 in the plant nucleus through BiFC assays, indicating that the EAR motif in SAN was crucial for recruiting the TPR2 corepressor complex to repress target genes. This interaction pattern aligns with the well-characterized mechanism of EAR-containing MYB transcription in *Arabidopsis*, such as MFS2, which recruited TPR1, TPR2 and TPR3 corepressors to suppress the expression of homeotic floral genes [36]. Our results extend this regulatory paradigm to rice anther development, establishing a SAN-TPR2 repressor complex as a key module governing cell division during anther growth. This finding advanced our mechanistic understanding of how SAN participated in the transcriptional modulation of downstream genes associated with cell cycle progression. The bona fide direct target genes bound and transcriptionally controlled by SAN remained to be experimentally identified. Collectively, these results indicated that SAN orchestrated a transcriptional cascade governing epidermal cell division during anther development. Given that most cell cycle- and cytokinin-related genes were downregulated in the san mutant, SAN likely acted indirectly on these genes—possibly by repressing upstream negative regulators of cell proliferation rather than directly targeting cell cycle genes themselves.

## 4. Materials and Methods

### 4.1. Plant Materials and Growth Conditions

The WT (wild-type) rice used in this study was in Nipponbare background (*Oryza sativa* L. spp. japonica). The *san* mutants were generated in the Nipponbare background using the CRISPR/Cas9 system (Peking University, Beijing, China). All plants were cultivated in greenhouse at 27–29 °C with a 14 h light/10 h dark photoperiod during winter and grown in paddy field during summer.

### 4.2. Vector Construction and Plant Transformation

The CRISPR/Cas9 vector *pUbi*-*Cas9* (provided by Prof. Lijia Qu, Peking University) [44] was used to construct the *SAN*-*CR1* and *SAN*-*CR2*. To generate highly specific single-guide RNAs (sgRNAs) with minimal off-target potential, two independent 20-nt target sequences ACAGCAGCGGCAGTAGTAGG (for *SAN*-*CR1*) and GCAGGCGAAGAAGGCGAGGT (for *SAN*-*CR2*) complied with the canonical SpCas9 NGG PAM requirement within the coding region of the *SAN* gene were rationally screened via the CRISPR-direct web server (http://crispr.dbcls.jp/ (accessed on 17 December 2019)) (Figure S1A–C). Rice mature seeds of Nip were cultured on solid N_6_ medium supplemented with 2,4-D for 4–5 weeks under dark conditions at 28 °C to induced calli. The two constructed editing vectors were individually introduced into healthy, compact yellow embryogenic calli via two completely independent batches of *Agrobacterium tumefaciens*-mediated genetic transformation assays using strain EHA105, to obtain genetically independent transgenic background lines. Putative transgenic plants (T_0_) were initially screened by selection on medium containing hygromycin. For the construction of *proSAN::GUS* and *proSAN::NLS*-*GFP* reporter vectors, an approximately 2.0 kb genomic fragment upstream of the *SAN* start codon, corresponding to the putative promoter region, was amplified and directionally cloned into the *pCAMBIA1301*-*GUS* vector and *PC1300G1*-*NLS*-*3×GFP*-*Nost* vector, respectively. For GUS staining and GFP fluorescence observation, 7 independent *proSAN::GUS* lines and 9 independent *proSAN::NLS*-*GFP* lines were generated through genetic transformation assays.

### 4.3. Phenotype Analysis of Anther

To analysis the phenotype of anthers, at least 20 florets of WT and *san* mutants were isolated from 3 independent plants. Anther was observed and photographed under stereomicroscope (SMZ25, Nikon, Tokyo, Japan) and the length of anther was measured using Digimizer 6.2.0 image analysis software (MedCalc Software Ltd., Ostend, Belgium). Anthers of WT and *san* mutants at different developmental stages corresponding to the P1–P6 stages of panicles were collected and fixed in FAA solutions. The P1–P6 stages were strictly referenced to the standard rice anther developmental to unify the developmental criteria for sample collection. P1 (0.3–3 cm young panicles) corresponds to the stamen primordium formation stage; P2 (3–6 cm panicles) is the four-lobed anther formation stage; P3 (6–10 cm panicles) represents archesporial cell differentiation, in which archesporial cells undergo periclinal division to produce primary parietal and primary sporogenous cells; P4–P5 (10–15 cm panicles) is the anther wall morphogenesis stage, forming a complete multi-layer anther wall and secondary sporogenous cells; P6 (15–20 cm panicles) is the microspore mother cell formation stage prior to meiotic initiation. Subsequently, anthers were subjected to whole-mount clearing and staining, stained with 0.1% Eosin B and cleared with methyl salicylate in order to observe the cellular development and anther epidermal cells via confocal laser scanning microscopy (Leica TCS SP8, Nidau, Switzerland). The length of 20 epidermal cells was taken as one data set, and epidermal cells from more than 20 anthers were measured using Digimizer software 6.2.0.

### 4.4. I_2_-KI and FDA Staining of Pollen

Pollen maturity and viability were assessed through I_2_-KI staining and fluorescein diacetate (FDA) staining respectively. For I_2_-KI staining, mature florets were placed in a drop of I_2_-KI solution and dissected anthers gently with forceps to release the pollen. The stained pollen grains were immediately observed under a light microscope (BX61, Olympus, Tokyo, Japan). FDA staining of pollen was performed similarly using freshly prepared FDA solution. Each experiment was conducted for 3 biological repeat from independent plant. For the count of pollens in WT and *san*, the single anthers of the floret were observed and used as the core biological statistical unit.

### 4.5. GUS Staining and GFP Fluorescence Observation

Tissues from *proSAN::GUS* transgenic plants were incubated in GUS staining solution according to the instructions provided in the GUS Staining Kit (GT0391-50S, Beijing Huayueyang Biotechnology Co., Ltd. Beijing, China) at 37 °C for overnight. Then, the stained tissues were cleared in 70% ethanol until the pigment of the tissues were discolored and imaged under a stereomicroscope (SMZ25, Nikon, Tokyo, Japan).

Anthers of *proSAN::NLS*-*GFP* transgenic plants at P1, P2–P3, P4, P5, and P6 stages were isolated and visualized using a confocal laser scanning microscope (TCS SP8, Leica, Nidau, Switzerland) to examine fluorescence expression in both WT and transgenic plants. The excitation wavelength was set at 488 nm, and the emission signal was collected within the range of 500–550 nm.

### 4.6. RNA Extraction, qRT-PCR and RNA-seq

Total RNAs was extracted from various tissues of rice, including seedlings, roots, stems, leaves, panicles at stages P1–P6, mature pistils, mature stamens, and seeds, using TransZol Plant Reagent (ET121, TransGen Biotech, Beijing, China). RNA was reverse-transcribed into cDNA using the EasyScript One-Step gDNA Removal and cDNA Synthesis SuperMix kit (AT311, TransGen Biotech, Beijing, China). Quantitative real-time PCR (qPCR) was performed on a CFX Connect Real-Time System (BIO-RAD, Hercules, CA, USA) with TransScript^®^ Top Green qPCR SuperMix (AQ131-01, TransGen Biotech, Beijing, China) using a modified method as described [45]. *Actin* was used as the internal reference gene. Each sample was analyzed with three biological replicates, and each biological replicate included three technical replicates. Relative gene expression levels were calculated using the 2^−ΔΔCt^ method.

For transcriptomic analysis, 100 mg mature anther of WT and *san*-*cr2*-*L1* mutant from 3 independent plants were respectively pooled as biological sample for isolation of total RNA. The cDNA libraries were synthesized from the mRNA purified using oligo dT and subjected to sequencing using an Illumina NovaSeq 6000 platform at Majorbio Co., Ltd. (Shanghai, China). The experiment included three biologically independent replicates. Differential expression analysis between WT and *san* mutant was carried out with DESeq2 [46], applying a threshold of q-value < 0.05 and log2 (fold change) ≥ 2 on TPM-normalized read counts. Functional enrichment analysis, including Gene Ontology (GO) and KEGG pathway assessment, was conducted via the OmicShare online suite (https://www.omicshare.com/tools, Guangzhou, China). Genes associated with selected biological processes were screened from the DEGs and, heatmaps were generated using TBtools v2.454 [47]. The validation of genes associated with the selected biological processes was conducted through qRT-PCR using 3 independent biological samples.

### 4.7. Subcellular Localization

To determine the subcellular localization of *SAN*, the full-length coding sequence (CDS) of *SAN* (without the stop codon) was cloned into the protein expression vector *pro35S::GFP* to generate the *pro35S::SAN*-*GFP* fusion construct. The empty *pro35S::GFP* vector was used as a control. Both constructs were separately transformed into rice protoplasts prepared from the sheaths and stems of etiolated seedlings (dark-grown for 10 days). After being transformed, the protoplasts were cultured in the dark at 28 °C for 16 h. The nucleus were stained with the nuclear specific dye 4′,6-diamidino-2-phenylindole (DAPI). Fluorescent signals were examined using a Leica SP8 confocal microscope (Nidau, Switzerland). GFP fluorescence was detected using an excitation wavelength of 488 nm and an emission window of 500–550 nm, while DAPI signals were captured with excitation at 405 nm and emission between 410 and 455 nm.

### 4.8. Sequence and Phylogenetic Analysis

Full-length amino acid sequences of *SAN* was downloaded from RGAP (https://rice.uga.edu/, Athens, GA, USA). The homologous proteins of SAN were retrieved from the NCBI database (https://www.ncbi.nlm.nih.gov/, Bethesda, MD, USA) for a broad range of species, encompassing angiosperms, gymnosperms, bryophytes, and metazoans. Multiple sequence alignment was conducted with ClustalW 2.1. The phylogenetic tree was constructed using the neighbor-joining method in MEGA7, with branch support evaluated by 1000 bootstrap replicates.

### 4.9. Transcription Activity Assay

The coding sequence of *SAN* fused with the GAL4 DNA-binding domain (GAL4BD) was cloned into the effector plasmid *pMpC1300*-*GAL4BD.* The reporter plasmid was constructed by inserting the firefly luciferase gene driven by the minimal 35S promoter into the upstream of the GAL4 binding sites. Both constructs were co-delivered into *Nicotiana benthamiana* leaves via *Agrobacterium tumefaciens*-mediated transient transformation. A 35S::Renilla luciferase construct was transfected as an internal control. Luciferase activities were measured 48 to 72 h after infiltration using the Dual-Luciferase^®^ Reporter Assay System (Promega, Madison, WI, USA). Firefly luciferase signals were normalized to the corresponding Renilla luciferase.

### 4.10. Bimolecular Fluorescence Complementation (BiFC) Assay

Bimolecular fluorescence complementation (BiFC) assays were performed using *pSPYNE*-*35S* (*YN*) and *pSPYCE*-*35S* (*YC*) BiFC vectors. The full length of *SAN* and *TPR2* were cloned into *YN/YC* vectors to generate fusions with the N-terminal (YN) or C-terminal (YC) fragments of yellow fluorescent protein (YFP). *SAN*-YN, *TPR2*-YC, *SAN*-YC, *TPR2*-YN, and empty vector controls plasmids *35S*-*SPYNE, 35S*-*SPYCE* were transformed into agrobacterium strain GV3101. Agrobacterial cultures were resuspended in infiltration buffer (10 mM MgCl_2_, 10 mM MES pH 5.6, 150 μM acetosyringone) and adjusted to an OD_600_ of 0.6 for each construct. The bacterial suspensions were co-infiltrated into the abaxial side of 4-week-old Nicotiana benthamiana leaves. Plants were cultured at 24 °C under 16 h light/8 h dark photoperiod for 48–72 h before fluorescence observation. YFP fluorescence signal was observed at 514 nm excitation and 522–555 nm emission in epidermal cells using a confocal microscope (TCS SP8, Leica, Nidau, Switzerland) for YFP detection. Three independent infiltration experiments were conducted, with at least three leaves infiltrated per construct combination in each replicate. Negative control combinations included YN/YC, SAN-YN/YC, SAN-YC/YN.

### 4.11. Statistical Analysis

All quantitative measurements were obtained from three separate biological samples. Two–tailed unpaired Student’s *t*-test (2-tailed) was performed to evaluate statistical differences. All statistical significance thresholds were defined as follows: *p* < 0.05 was considered statistically significant, and *p* < 0.01 represented extremely significant differences. Graph plotting and all statistical calculations were completed with GraphPad Prism 9 software.

## 5. Conclusions

In summary, this study identified *SAN* as a novel and specific regulator of rice anther length. Loss of *SAN* function led to specific anther shortening and reduced pollen number, without impairing pollen viability or other major agronomic traits. *SAN* encoded a nucleus-localized C1-1iG-type zinc finger protein harboring a conserved EAR motif and showed strong transcriptional repression activity. This repression likely occured through the recruitment of the corepressor TPR2, forming a functional transcriptional repression complex. Transcriptomic and molecular analyses revealed that *SAN* modulated the expression of core cell cycle, cell wall biosynthesis, and hormone signaling genes to control cell proliferation. These findings expanded the molecular basis of anther size control in rice and provided a valuable genetic resource for restorer line in hybrid rice seed production.

## Data Availability

Sequence data from this article can be found in the RGAP data libraries under the following accession numbers: LOC_Os02g01090. All raw transcriptomic sequencing reads have been deposited into the NCBI Sequence Read Archive (SRA) database under the BioProject accession PRJNA1492215.

## Supplementary Materials

The following supporting information can be downloaded at: https://www.mdpi.com/article/10.3390/plants15152246/s1, Figure S1: The CRISPR/Cas9 target design and off-target prediction; Figure S2: Phenotype characteristics of WT and san mutants during vegetative development; Figure S3: Phenotype and statistical analysis of panicle in *san* mutants; Figure S4: Expression levels of *SAN* in different tissues of rice; Figure S5: The phylogenetic analysis of SAN; Table S1: The primers used in this study.
