# Supplementary material for: Small Anther, a C2H2-Type Zinc Finger Transcription Repressor, Controls Rice Anther Length Through Regulation of Epidermal Cell Division
Source: Plants (Basel). 2026 Jul 23;15(15):2246. doi: 10.3390/plants15152246 (PMC13468419; doi:10.3390/plants15152246)
Supplement: Supplementary file 1 [file plants-15-02246-s001.zip › plants-4407451-supplementary.pdf]

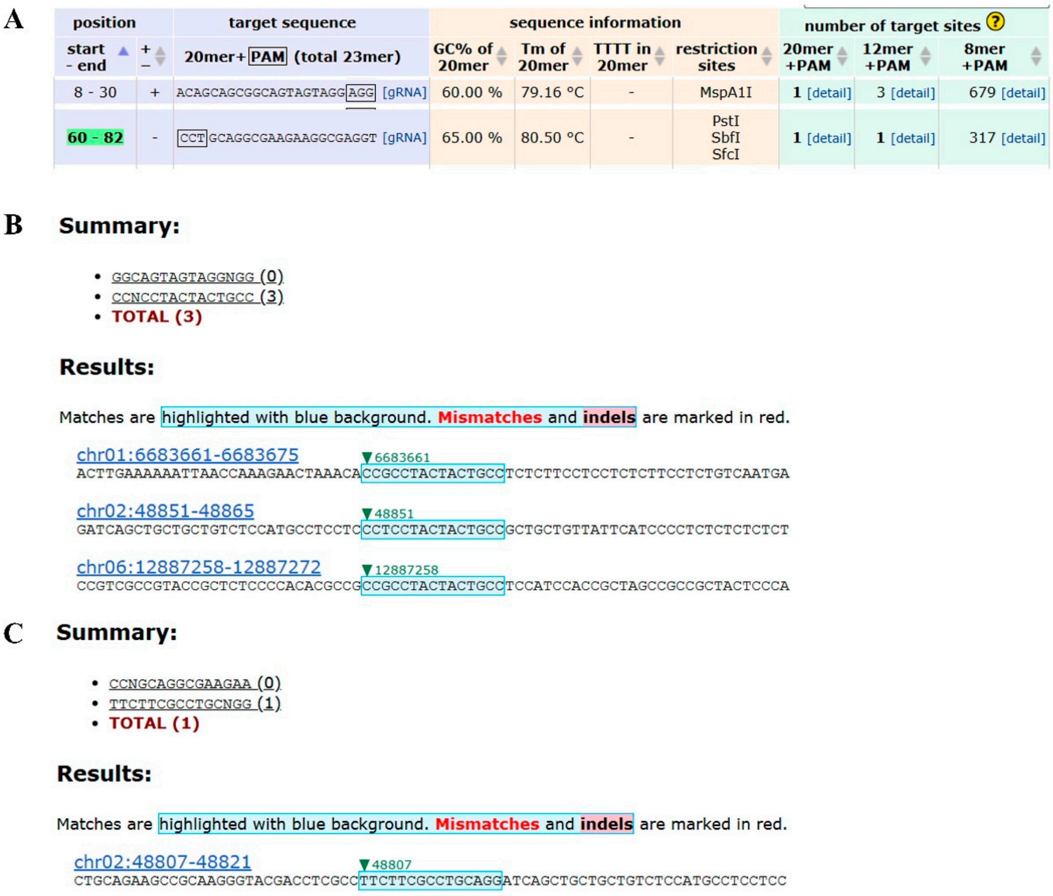

Figure S1. The CRISPR/Cas9 target design and off-target prediction. (A) The target design of *SAN–CR1* and *SAN–CR2* by CRISPR-direct. (B) The off–target prediction of *SAN–CR1*. (C) The off–target prediction of *SAN–CR2*.

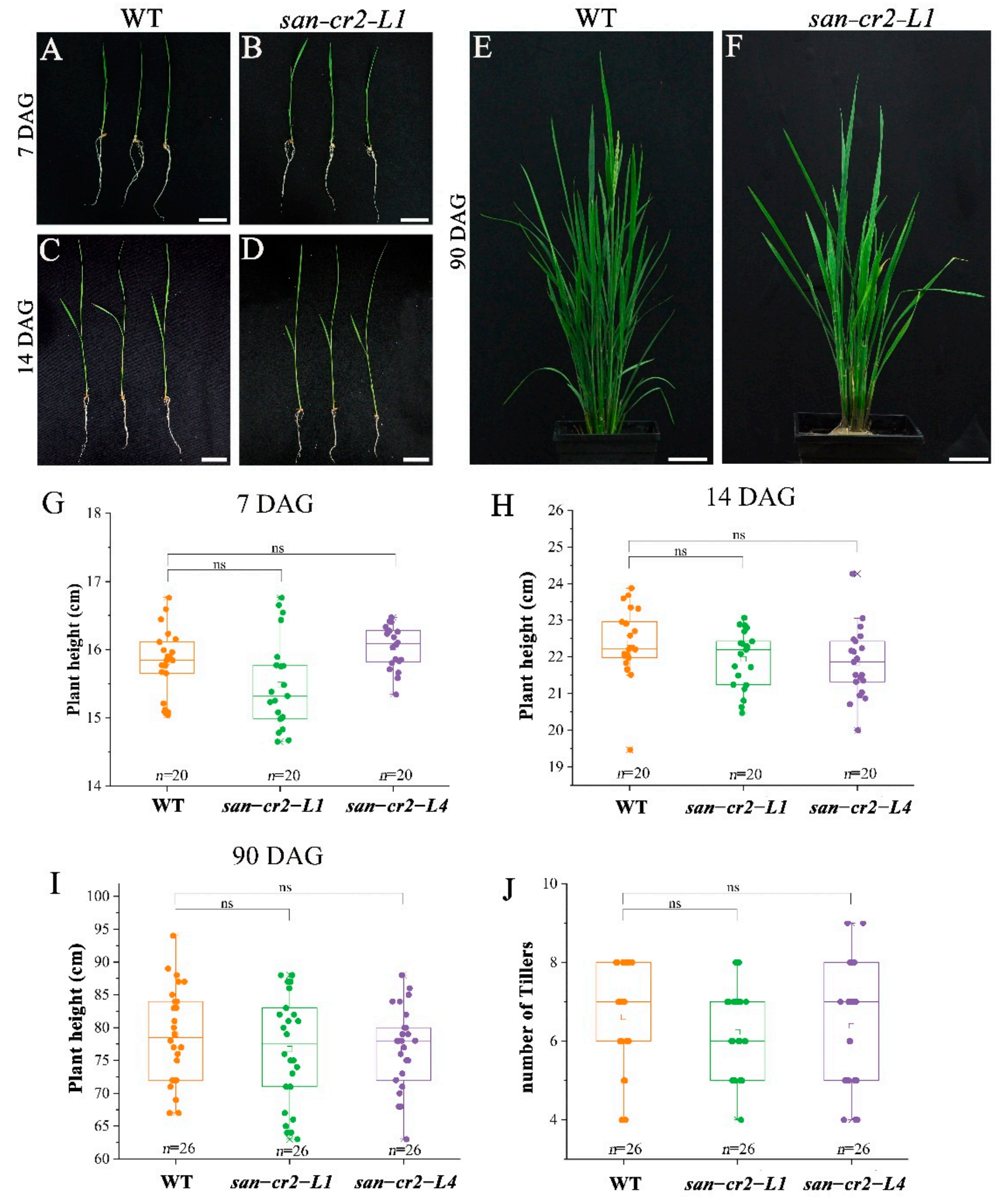

Figure S2. Phenotype characteristics of WT and *san* mutants during vegetative development. (A-B) The observation of 7−dyas−old seedlings of WT and *san* mutants. Bars = 5cm. (C-D) The observation of 14−dyas−old seedlings of WT and *san* mutants. Bars = 5cm. (E-F) The observation of 90−dyas−old seedlings of WT and *san* mutants. Bars = 4cm. (G-J) The statistic analysis of height and tiller numbers of WT and *san* mutants in 7−days−old seedlings, 14−days−old seedlings and 90−days−old seedlings. DAG, day after germination. ns represents no significant difference.

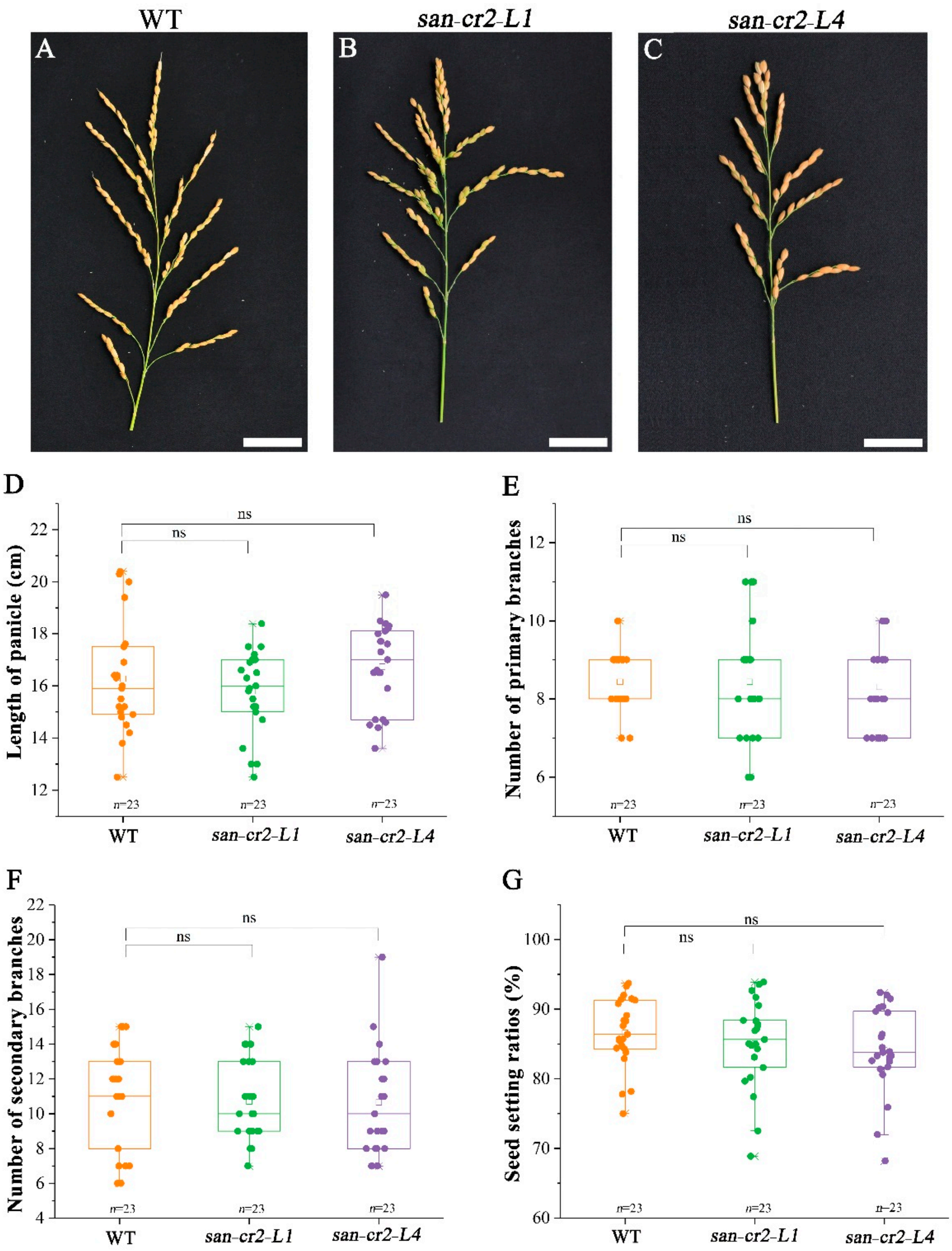

Figure S3. Phenotype and statistical analysis of panicle in *san* mutants. (A–C) The mature panicle of WT, *san−cr2−L1* and *san−cr2−L4*. The scale bars = 4 cm. (D) The length of panicles in WT and *san* mutants. (E) The number of primary branches in WT and *san* mutants. (F) The number of secondary branches in WT and *san* mutants. (G) The seed setting ratios of WT and *san* mutants. ns represents no significance.

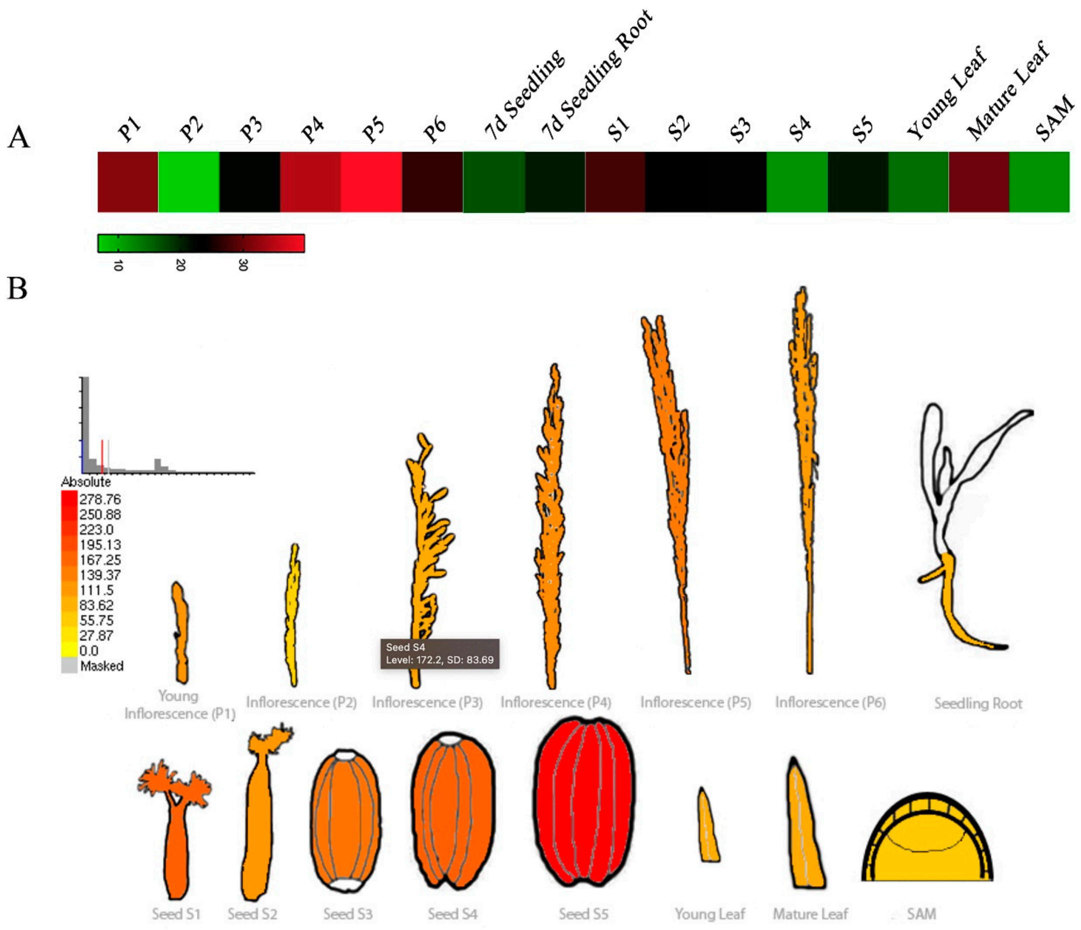

Figure S4. Expression levels of *SAN* in different tissues of rice. (A) Expression patterns of *SAN* in different tissues of rice analysed by the microarray database. (B) Expression patterns of *SAN* in different tissues of rice from the BAR website. Young Inflorescence at different developmental stages: P1, 0–3 cm; P2, 3–5 cm; P3, 5–10 cm; P4, 10–15 cm; P5, 15–22 cm; P6, 22–30 cm. Seeds at different stages after pollination: S1, 0–2 d; S2, 3–4 d; S3, 5–10 d; S4, 11–20 d; S5, 21–29 d.

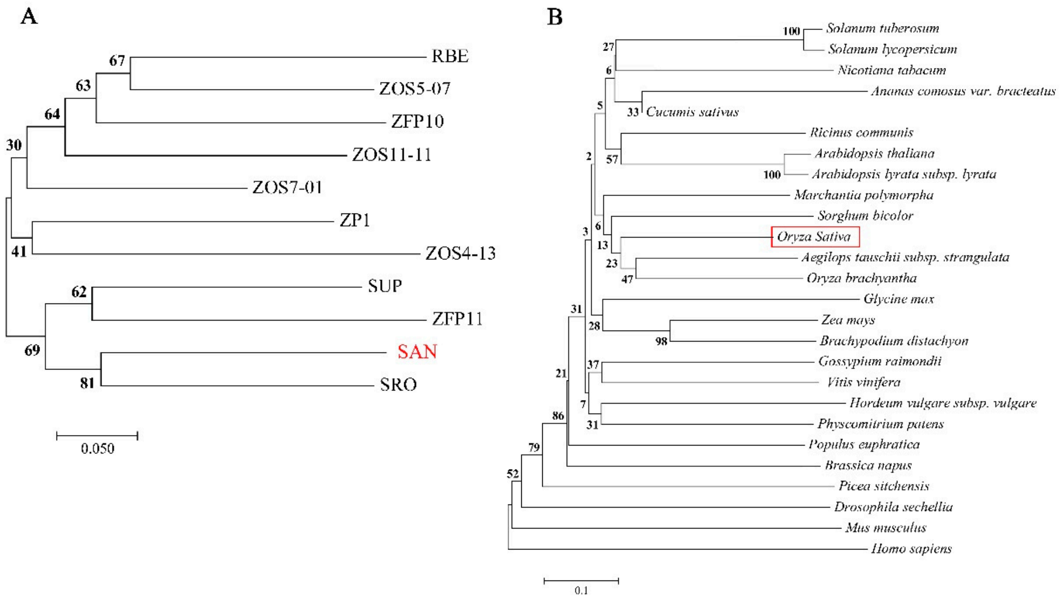

Figure S5. The phylogenetic analysis of SAN. (A) Evolutionary tree analysis of the member of C1−liG subgroup in rice and Arabidopsis. (B) Evolutionary tree analysis of SAN in rice and other homologous proteins from various species.

Table S1. The primers used in this study

Primers for the construct and identification of CRISPR-Cas9 vectors

Primers name	Primers sequence
01090-cr1-F	GGCAGCAGGCGAAGAAGGCGAGGT
01090-cr1-R	AAACACCTCGCCTTCTTCGCCTGC
01090-cr2-F	GGCAACAGCAGCGGCAGTAGTAGG
01090-cr2-R	AAACCCTACTACTGCCGCTGCTGT
Ubi-RP	GATAAACTGCACTTCAAACA
PC1300-RP	AGGCACCCCAGGCTTTACACT

Primers for subcellular location fusion construct

Primers name	Primers sequence
01090-CDS-EGFP-FP	CGGGATCCATGAATAACAGCAGCGGC
01090-CDS-EGFP-RP	GGAATTCGAAGAAGGCGAGCCTAAGCTC
Pro01090-GUS-FP	CGGGATCCATAGCCCTGCACATACCG
Pro01090-GUS-RP	AACTGCAGCATCCCCTCTCTCTCTCTCTCTCTCTCTCTCGCTCTCG CTCTGGATATC
pro01090-EGFP-FP	AACTGCAGATAGCCCTGCACATACCG
pro01090-EGFP-RP	CGGGATCCCCCCTCTCTCTCTCTCTCTCTCTCTCTCGCTCTCGCTC TGGATATCAATAGCTACTAC

Primers for qRT-PCR

Primers name	Primers sequence
13860-qF	TGGACACAGTGGCATAATCG
13860-qR	ACCGCACCTTCTTGAGAGC
37390-qF	GGAGGTTTACACTTACTACAACTTCA
37390-qR	CGTGGTTCCCCAATCTGA
10256-qF	GCCTCGTCATCATCCGCT
10256-qR	GCTGCCAACACCTCATCCA
47870-qF	CTGCCGCAACCACATCAT
47870-qR	TTCTGGAACTCCCACTCGC
13850-qF	AGATGTGGATTCGTTTCCTATG
13850-qR	GTCAGCAACTGTCCGCACT
47580-qF	TTGACCATCAGGTTTCCGT
47580-qR	TCATCCTCATCAGTATCATTGC
50140-qF	CGGCTCATCCTCACTACGGT
50140-qR	GAGAGAATGGAGATTGATTTGGCTA
09660-qF	GGTTATCCCTCTCTTCCGTC
09660-qR	GTGGTTGCATGGTAAAGTCTAGT
37370-qF	GGCAAAGAAGTGGCAACAT
37370-qR	CCTAAGCAAGCACATCACATAC
32680-qF	CGTGCGATTGACGGTGTGA
32680-qR	TTGAGGTAGCCGAGTGGGTTG
27710-qF	CCATCGTGGTATCAGCCGT

27710-qR	TGTAGCAAGTTTCTTCTCATCTCC
05710-qF	GCCAATACTCCTTCGCCTT
05710-qR	GACATCGGTTGATCCTCCTG
22210-qF	ATCAACCTGGGGAAGCTCA
22210-qR	CGATGGGACGATGCTAATG
OsActin1-qF	AGCAGCATGAAGATCAAGGTGGTC
OsActin1-qR	CCTTGGCAATCCACATCTGCTG
